# *Biomphalaria camerunensis* as a viable alternative intermediate host for *Schistosoma mansoni* in southern Cameroon

**DOI:** 10.1186/s13071-018-2763-2

**Published:** 2018-03-13

**Authors:** Alvine C. Kengne-Fokam, Hugues C. Nana-Djeunga, Mohamed Bagayan, Flobert Njiokou

**Affiliations:** 1 Parasitology and Ecology Laboratory, Faculty of Science, University of Yaoundé 1, PO Box 812, Yaoundé, Cameroon; 2 Center for Research on Filariasis and other Tropical Diseases (CRFilMT), PO Box 5797, Yaoundé, Cameroon; 3 Laboratory of Animal Biology and Ecology, University of Ouaga I Pr Joseph Ki-Zerbo, Ouagadougou, Burkina Faso; 4 Health Science Research Institute (IRSS), Ouagadougou, Burkina Faso

**Keywords:** *Biomphalaria pfeifferi*, *Biomphalaria camerunensis*, *Schistosoma mansoni*, Compatibility, Cercarial production, Cameroon

## Abstract

**Background:**

Intestinal schistosomiasis due to *Schistosoma mansoni* was mapped in Cameroon in the 1990s and preventive chemotherapy launched since 2005. A situation analysis conducted in 2011 revealed an increase in schistosomiasis transmission, especially in the equatorial part of the country, despite the fact that *Biomphalaria pfeifferi*, the main intermediate host of this parasite, is now scarce in many foci. *Biomphalaria camerunensis*, restricted to the equatorial part of the country, is considered as a less suitable host for *S. mansoni* due to it resistance to the parasite, although exhibiting a better survival than *B. pfeifferi*. In a context where human migration is quite frequent as a consequence of terrorism, war in neighboring countries, as well as development of hydraulic projects, it seems appropriate to evaluate the current epidemiological role of *B. camerunensis* to estimate the risk of extension of *S. mansoni* distribution in Cameroon. To do this, the susceptibility of three *B. pfeifferi* and five *B. camerunensis* populations to a strain of *S. mansoni* was assessed. Juvenile snails (G1) of each population were infected with *S. mansoni* miracidia, and prepatent period, infection and survival rates of infected snails, as well as cercarial production were recorded and compared between snail species and populations.

**Results:**

Compatibility tests were performed on a total of 827 snails: 344 *B. pfeifferi* and 483 *B. camerunensis*. Infection rates were quite heterogeneous, higher in *B. pfeifferi* (61.5%) as compared to *B. camerunensis* (7.8%) (Chi-square test: *χ*^2^ = 258.88, *df* = 1, *P* < 0.0001). All the three *B. pfeifferi*-infected populations were susceptible to *S. mansoni*. However, among the five *B. camerunensis* populations tested, four were susceptible to *S. mansoni*, with 21.9% as the highest infection rate. The prepatent period was, on average, shorter in *B. pfeifferi* than in *B. camerunensis* (*P* < 0.0001), but the cercarial production was significantly higher in *B. camerunensis* as compared to *B. pfeifferi* (*P* < 0.001).

**Conclusion:**

These findings indicate that *B. camerunensis* populations might substantially contribute to *S. mansoni* transmission and dissemination in Cameroon, their low susceptibility being compensated by their high cercariae production. More attention and surveillance towards this species are required to achieve intestinal schistosomiasis elimination in Cameroon.

**Electronic supplementary material:**

The online version of this article (10.1186/s13071-018-2763-2) contains supplementary material, which is available to authorized users.

## Background

Schistosomiasis has been known to be endemic in Cameroon for more than three decades [1, 2]. Control activities started with the creation of the National Program for the Control of Schistosomiasis and Soil Transmitted Helminthiasis (PNLSHI) in 2003, and have been rapidly scaled up. In addition to the mass administration of praziquantel to school-aged children (5–14 years) since 2005, the schistosomiasis control strategy in Cameroon also relies on education of communities about the disease and risks of infection, and the promotion of hygiene and environmental sanitation. These actions have contributed to significantly reducing the prevalence of this disease in several foci [3, 4]. Despite these control measures, the current epidemiological situation reveals the persistence of schistosomiasis in some foci, and the emergence of new foci of the disease [3–5]. The establishment of new foci could be explained by the migration of parasitized individuals to disease free sites where sanitation is inadequate and intermediate hosts present [6]. To efficiently interrupt schistosomiasis transmission, the WHO recommends a synergy of actions, among which the fight against intermediate hosts holds a prominent place [7].

Intestinal schistosomiasis due to *Schistosoma mansoni* is the most largely distributed form of schistosomiasis in equatorial zones of Cameroon. *S. mansoni i*s transmitted by two snails species of the genus *Biomphalaria* (*B. pfeifferi* and *B. camerunensis*), which present a wide geographical distribution though *S. mansoni* remains focalized. It was shown that *B. pfeifferi* preferentially reproduced by self-fertilization [8, 9], thus exhibiting low genetic diversity within populations, and low survival of the offspring [9]. It is the main *S. mansoni* host in sub-Saharan Africa [10, 11], including Cameroon where the most active *S. mansoni* foci are maintained by this snail species. This intermediate host is, however, becoming scarcer in many sites in Cameroon where it used to be abundant, likely due to climatic and environmental changes operating as a consequence of global warming. *B. camerunensis* species is expected to present a more polymorphic structure and a better offspring survival as an advantage of its preferential outbreeding reproduction system [9, 12]. This species is found in the equatorial bioclime zone of the country, and was previously described as poorly susceptible to *S. mansoni* [11]. Two *B. camerunensis* sub-species are known to prevail in Central Africa, *B. camerunensis camerunensis* found in many localities in Cameroon, and *B. camerunensis manzadica* described in Bas-Congo in Democratic Republic of Congo (DRC) [13]. The latter species was shown to be involved in the creation and maintenance of *S. mansoni* active foci in DRC [14], and according to Frandsen [10], some *B. camerunensis* populations are as susceptible to *S. mansoni* as *B. pfeifferi* populations.

In Cameroon, human migration is quite frequent, mainly as a consequence of job seeking and instability related to wars and terrorism. The most important migration flow is originated from the Far North Region and from neighboring countries (Nigeria, Chad, Central African Republic) where *S. mansoni* is highly endemic, toward the South Region where many structuring projects (dams, ports and road construction) are ongoing. Indeed, a relatively high prevalence of onchocerciasis has been found in migrants escaping civil war from Central African Republic and who have now settled in the East Region of Cameroon), raising the concern of cross-border transmission of this filarial infection (Nana Djeunga et al., personal communication).

The aim of this study is to investigate the susceptibility of different *B. camerunensis* populations to *S. mansoni*, in order to assess the risk of establishment of new intestinal schistosomiasis foci as a corollary of potential introduction of foreign parasite in areas where this snail species is largely distributed.

## Methods

### Snail collection and maintenance

**Fig. 1:**
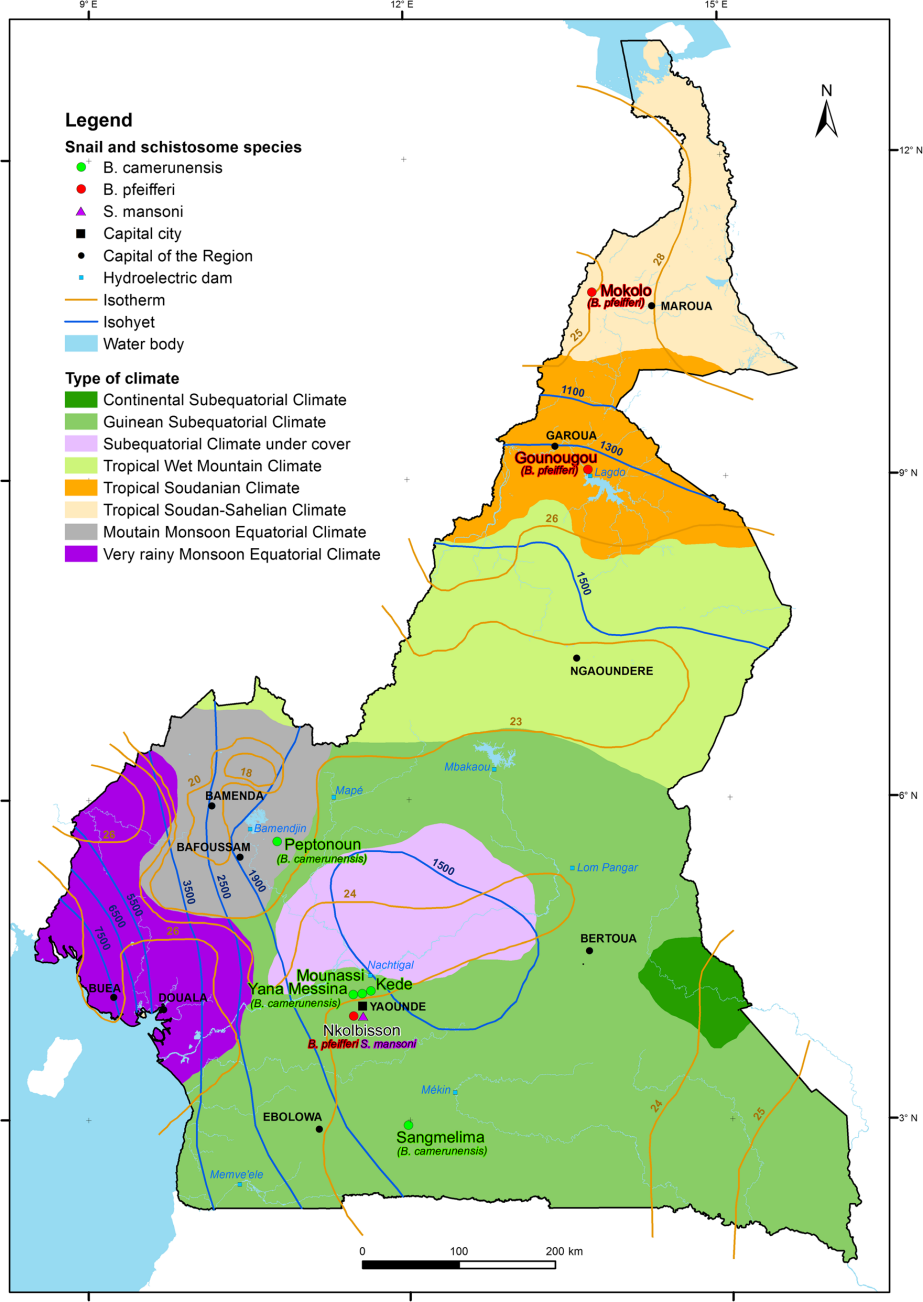
Location of snails and parasites sampling sites according to climatic zones and temperature delineations in Cameroon

Snails were collected in eight sites (Fig. 1), using either a stainless steel sieve mounted on a 1.5 m wooden handle to comb aquatic vegetation, or by hand picking from the mud. *Biomphalaria pfeifferi* snails were collected in three sites, namely Ekorezok (3°53'327"N, 11°27'412"E) in the Center Region, Gounougou (9°4'33"N, 13°42'25"E) in the North Region, and Mokolo (10°44'00"N, 13°46'4"E) in the Far North Region. *B. camerunensis* snails were collected in five sites among which three are located in the Centre Region [Mounassi (4°12'14.1"N, 11°35'0.2"E), Kede (4°12'6"N, 11°35'6"E), and Yana Messina (4°12'1"N, 11°35'3"E)], one in the South Region [Sangmelima (2°56'24.6"N, 11°58'34.7"E)] and one located in the West Region [Petponoun (5°37'59.12"N, 10°38'7.35"E)].

**Fig. 2:**
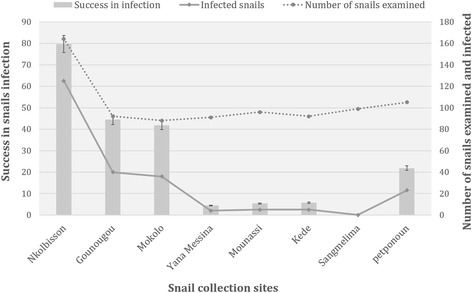
Success in snail infection according to collection sites. The dashed line represents the number of snails examined, the solid line represents the number of snails infected and the grey rectangle represents the percentage of snails infected in each collection site

Once in the laboratory, snails collected were sorted (based both on their shell morphology [15] and polymerase chain reaction restriction fragment length polymorphism analysis (PCR-RFLP) of the ITS2 region [16]) and distributed individually in 100 ml well labeled plastic boxes containing spring water. A piece of polystyrene was added in each rearing box for eggs laying. Overall, 15 sexually mature wild snails were isolated from each of the eight populations. One to two series of egg-laying were collected from each individual snail. After hatching, offspring were reared separately for one month [9, 17]. This protocol was therefore important to monitor the number of wild snails contributing to the constitution of offspring samples to be exposed to miracidia.

### Acquisition of schistosomes

A parasitological survey was performed in the Nkolbisson quarter (Ekorezok site), located in Yaounde (Cameroon City Capital), at about 7 km from the central town. Eligible individuals were those living near the Afeme River and who have been regularly in contact with the infested water of the river as a consequence of their professional and household activities. Stool samples were collected from each volunteer and analyzed in the laboratory using the Kato-Katz technique [18]. A total of nine stool samples with an intensity of infection ranging between 200 and 1700 eggs per gram of stool (epg) were used for parasite acquisition. Eggs were obtained after a series of washing and sedimentation of feces using saline solution, then spring water. Isolated eggs hatched under the action of both osmotic and thermal shock. Indeed, the pellet obtained after sedimentation was exposed to an artificial source of light for 30 min. After hatching, these eggs released miracidia necessary for the infection of the juvenile snails.

### Snails’ infection and follow-up of compatibility metrics

For each population, two to six young snails (2–4 mm), originating from each of the 15 wild parents, were individually put in the wells of a microtiter plate containing spring water. Five miracidia were then pipetted under a binocular dissecting microscope, and transferred in a well containing a young snail. This exposition of young snails to parasites (miracidia) was performed at room temperature (26 ± 1 °C) for 4 h. A total of 827 snails (344 and 483 of *B. pfeifferi* and *B. camerunensis*, respectively) were exposed to the parasite. Each potentially infected offspring was returned back to its rearing box. Between 20 and 60 days post-infection, infected snails were followed up daily for cercarial shedding. The prepatent period (the duration of the larval development up to the first cercarial shedding) and the number of snails surviving at day 60 post-infection were recorded. The infection rate of each population was estimated as the ratio of the number of living individuals producing cercariae on the number of exposed living individuals.

Cercarial production was assessed from 40 days post-infection, when most of the potentially infected snails were susceptible to start shedding cercariae. Cercarial production was followed up for five consecutive days, for 16 and 8 infected individuals belonging to *B. pfeifferi* (from Nkolbisson) and *B. camerunensis* (from Petponoun), respectively. Snails were kept individually at room temperature (26 ± 1 °C) for 24 h in 20 ml of water, and cercariae were counted under a dissecting microscope in a checkered plate containing 5 ml of this water and one or two drops of Lugol acting as a fixative for the cercariae.

### Data analysis

Relevant data were entered into a purpose-built Microsoft Office Excel dataset, and analyzed using both the PASW Statistics Version 18 (SPSS Inc., Chicago, IL, USA) software, and the online program Vassarstat statistical computational web site [19], the latter being particularly useful for the calculation of the 95% confidence intervals. The mortality and infection rates were calculated and then compared between the different snail populations using the Chi-square test; Yates correction for continuity and Fisher's exact tests were used for small sample sizes (calculated or expected value Although a normal distribution was not expected for count data, the normality of distributions was tested and non-parametric tests chosen to analyze quantitative data. The non-parametric Mann-Whitney rank test was used to compare the duration of the prepatent period between species as well as the cercarial production between the different days of follow-up, and the two species. The non-parametric Kruskall-Wallis test was used to compare the duration of the prepatent period between snails populations, and pairwise comparisons performed when the difference was significant. The threshold for significance was set at 5% for all tests.

## Results

A total of 344 *B. pfeifferi* and 483 *B. camerunensis* snails were exposed and followed up to investigate the compatibility between *Biomphalaria* spp. and *S. mansoni*.

### Infection rates

The infection rates were quite heterogeneous (Table 1). Indeed, they were found to differ both between infected populations (Chi-square test: *χ*^2^ = 325.47, *df* = 7, *P* < 0.0001) and snail species (*B. pfeifferi* and *B. camerunensis*) (Fig. 2). The comparison of infection rates between the two snail species revealed that *B. pfeifferi* was significantly more infected (61.5%) than *B. camerunensis* (7.8%) (Chi-square test: *χ*^2^ = 258.88, *df* = 1, *P* < 0.0001).

**Table 1:** Mortality and success of infection rates of snails during prepatent and patent periods

Snail species	Snail population	Prepatent period						Patent period		
		No. of snails exposed	No. of surviving snails	Mortality rate (%)	No. of infected snails	Infection rate (%)	Duration of pp	No. of surviving snails	No. of infected snails surviving	Mortality rate (%)
*B. pfeifferi*	Nkolbisson	164	157	4.3	125	79.6	21–25	115	110	29.9
	Gounougou	92	90	2.2	40	44.4	25–32	66	22	28.3
	Mokolo	88	80	2.3	36	41.9	25–40	50	6	43.2
	Total	344	327	4. 9	201	61.5		231	138	32.8
*B. camerunensis*	Yana Messina	91	90	1.1	4	4.4	34–36	63	4	30.8
	Mounassi	96	93	3.1	5	5.3	34–40	78	4	18.8
	Kede	92	87	5.4	5	5.7	34–40	75	4	18.8
	Sangmelima	99	97	2.0	0	0.0	0	81	0	18.2
	Petponoun	105	105	0.0	23	21.9	28–32	21	8	80.0
	Total	483	472	2.3	37	7.8		318	20	34.2

*Abbreviations*: *SIR*, success of infection rate, *pp* prepatent period

Regarding *B. pfeifferi*, all the three populations investigated were susceptible to the parasite (*S. mansoni*), the highest infection rate (79.6%) being found in the site where the parasites were originated from (Nkolbisson). The infection rates in sympatric population were significantly higher than those found in allopatric populations from Gounougou (Chi-square test: *χ*^2^ = 31.91, *df* = 1, *P* < 0.0001) and Mokolo (Chi-square test: *χ*^2^ = 35.43, *df* = 1, *P* < 0.0001).

As for *B. camerunensis*, four populations amongst the five tested were susceptible to *S. mansoni*. The highest infection rate (21.9%) was found in the Petponoun population, and was significantly different from those found in the Yana Messina (4.4%) (Chi-square test: *χ*^2^ = 12.39, *df* = 1, *P =* 0.0004), Mounassi (5.4%) (Chi-square test: *χ*^2^ = 11.10, *df* = 1, *P =* 0.0009) and Kede (5.7%) (Chi-square: *χ*^2^ = 9.97, *df* = 1, *P =* 0.0016) populations.

Pairwise comparisons of infection rates among populations, for both *B. pfeifferi* and *B. camerunensis*, are given as supplementary material (Additional file 1: Table S1).

### Mortality rates

At the end of the prepatent period (day 60 post-infection), the mortality rate in snails was similar both between the species (4.9 and 2.3% in *B. pfeifferi* and *B. camerunensis*, respectively) (Chi-square test: *χ*^2^ = 0.200, *df* = 1, *P* = 0.655), and the populations, either for *B. pfeifferi* (Chi-square with Yates correction for continuity: *χ*^2^ = 4.09, *df* = 2, *P* = 0.1294) or for *B. camerunensis* (Chi-square with Yates correction for continuity: *χ*^2^ = 7.48, *df* = 4, *P* = 0.1126).

During the patent period, a high mortality rate was observed in the different populations. Although remaining similar between the two snail species (Chi-square test: *χ*^2^ = 0.359, *df* = 1, *P* = 0.549), the mortality rates observed were significantly different between both *B. pfeifferi* populations (Chi-square test: *χ*^2^ = 128.18, *df* = 2, *P* < 0.0001) and *B. camerunensis* populations (Chi-square with Yates correction for continuity: *χ*^2^ = 41.78, *df* = 4, *P* < 0.0001) (Table 1). The mortality rate among *S. mansoni-*infected individuals was significantly higher in allopatric populations (Mokolo and Petponoun) as compared to their rates in the sympatric population (Nkolbisson) to the parasite. Pairwise comparisons of mortality rates among populations, either *B. pfeifferi* or *B. camerunensis*, are given in Additional file 2: Table S2.

### Duration of the prepatent period

The duration of the prepatent period varied from 21 to 40 days (Mean: 22.51, SD: 1.945) and from 28 to 40 days (Mean: 29.65, SD: 3.442) for *B. pfeifferi* and *B. camerunensis* populations, respectively (Table 1). It was significantly different both between the two snail species (Mann-Whitney U-test: *U* = 0.000, *Z* = -10.66, *P* < 0.0001), and between the *B. pfeifferi* populations (Kruskal-Wallis H-test: *χ*^2^ = 129.327, *df* = 2, *P* < 0.0001). As regards to *B. camerunensis*, the duration of the prepatent period was similar among the populations from Yana Messina, Mounassi and Kede (Kruskal-Wallis H-test: *χ*^2^ = 0.390, *df* = 2, *P* = 0.821), but was significantly lower in the snail population originating from Petponoun as compared to those of Kede (Mann-Whitney U-test: *U* = 0.000, *Z* = -5.20, *P* < 0.0001), Mounassi (Mann-Whitney U-test: *U* = 0.000, *Z* = -5.20, *P* < 0.0001) and Yana Messina (Mann-Whitney U-test: *U* = 0.000, *Z* = -5.10, *P* < 0.0001). The duration of the prepatent period was negatively correlated with the infection rate (Spearman's rank correlation test, *r* = -0.847, *P* = 0.016).

### Cercarial production

Table 2 summarizes the cercarial production follow-up for the two *Biomphalaria* species. The cercarial production was similar in both snail species on day 1 (Mann-Whitney U-test: *U* = 41.00, *Z* = -1.41, *P* = 0.158), but significantly more important in *B. camerunensis* than in *B. pfeifferi* from day 2 onwards (Mann-Whitney U-test: *U* = 7.00, *Z* = -2.67, *P* < 0.008) (Table 2). Some of the infected *B. pfeifferi* survived more than eight months post-exposure while continuing to shed cercariae. The mean value of daily cercarial production in *B. camerunensis*/*S. mansoni* combination was 1968.38 cercariae. Globally, cercarial production over five days of follow-up was significantly more important in *B. camerunensis* than in *B. pfeifferi* (Mann-Whitney U-test: *U* = 9.00, *Z* = -3.37, *P* = 0.001).

**Table 2:** Follow-up of the cercarial production in *B. pfeifferi* of Nkolbisson and *B. camerunensis* of Petponoun over five days

Follow-up day		*n*	Mean ± SD	Minimum	Maximum	*P*-value
Day 1	*B. pfeifferi*	16	1376.5 ± 906.2	50	3874	0.158
	*B. camerunensis*	8	2143.4 ± 1381.2	850	4900	
Day 2	*B. pfeifferi*	16	588.8 ± 419.1	8	1288	0.001
	*B. camerunensis*	8	1879.0 ± 639.6	520	2676	
Day 3	*B. pfeifferi*	16	1086.1 ± 542.1	8	2380	0.001
	*B. camerunensis*	8	2063.1 ± 404.9	1208	2564	
Day 4	*B. pfeifferi*	16	1074.2 ± 634.8	12	2160	0.008
	*B. camerunensis*	8	1916.5 ± 564.4	1012	2564	
Day 5	*B. pfeifferi*	16	1245.4 ± 514.1	40	2068	0.001
	*B. camerunensis*	8	2212.9 ± 538.9	1040	2780	
Total	*B. pfeifferi*	16	1074.2 ± 454.4	23.6	1925.6	0.001
	*B. camerunensis*	8	2042.9 ± 485.2	1073.6	2633.6	

*Abbreviation*: *SD* standard deviation

## Discussion

This study was conducted in Cameroon in a context where *S. mansoni* transmission is persisting, with the creation of new foci [3, 4]. This situation might have been driven by a number of reasons [18], and adaptation of a ‘foreign’ parasite to an intermediate host appears essential to the creation of new foci for disease expansion.

## Compatibility

The infection rate was quite heterogeneous in the present study, with differences observed both between the two species and within the same species, thus confirming the important polymorphism of the susceptibility of *Biomphalaria* species [10, 20–22]. These results likely indicate an important genetic diversity among snail populations as was previously observed in Cameroon [12] and Madagascar [23]. The highest infection rate was observed in the sympatric pair *B. pfeifferi*/*S. mansoni* of Nkolbisson, suggesting that this parasite is best adapted to its local snail population as previously described [15, 22], though local adaptation is not always the rule [24]. This parasite strain seems to be less adapted to the population of northern Cameroon (Gounougou and Mokolo) as supported by the significantly high mortality rate in *S. mansoni-*infected snails belonging to these populations. This could be explained by the quite different conditions (climatic and phytogeographical) prevailing in these areas compared to that where the parasite is originated from (Fig. 1).

Most of the *B. camerunensis* populations exhibited low infection rates (one being totally refractory) to the *S. mansoni* strain from Nkolbisson. This observation might suggest that these populations are resistant to the penetration and development of *S. mansoni* as a consequence of genetic predisposition of these snails to destroy the parasite as a foreign entity. A similar result was obtained by Simões et al. [6] in populations of *Biomphalaria taenagophila* and *B. straminea*, intermediate hosts of *S. mansoni* in Brazil. However, the Petponoun population exhibited a relatively high susceptibility rate (21.9%), quite similar to that of other *B. pfeifferi* [10], *B. glabrata* (the main host of intestinal schistosomiasis in Brazil) [25] and *B. taenagophila* [26] populations. This result suggests that some *B. camerunensis* populations could be as susceptible to *S. mansoni* as their ‘appropriate’ intermediate hosts, contrary to the until now accepted idea that *B. camerunensis* is a poor host with almost no role in the transmission of *S. mansoni*. Despite this high susceptibility, the significantly high mortality rate observed in infected individuals from Petponoun (Table 1) reflects the adverse effect of parasitic development on the survival of these infected individuals, and suggests that adaptation of the Petponoun population to *S. mansoni* from Nkolbisson is far from perfect. Since susceptibility to *S. mansoni* is easier to obtain over generations than resistance [26], and that an increase in temperature can induce the inversion of resistance to *S. mansoni* [27], the susceptibility of *B. camerunensis* populations to *S. mansoni* may increase as a consequence of global warming. Indeed, in Cameroon, the snail *Bulinus camerunensis* which has been formerly known to be resistant to *S. haematobium* strain of Barrombi-kotto is now susceptible to that strain [28]. Also, in southern Corsica (France), the susceptibility of *Bulinus truncatus* to a hybrid schistosome (*S. haematobium*/*S. bovis*) has contributed to the re-emergence of schistosomiasis [29–31].

The duration of the prepatent period was negatively correlated with the infection rate and was quite different between snail populations (either *B. pfeifferi* or *B. camerunensis*), suggesting that a short prepatent period reveals a better compatibility to the parasite and that our populations are genetically different. Interestingly, the cercarial production was two-fold higher in *B. camerunensi*s than in *B. pfeifferi*, though this observation was done only in one *B. camerunensis* population among the five investigated. Indeed, Bennike et al. [32] also reported a high and constant cercarial production in susceptible *B. camerunensis manzadica* individuals, as compared to susceptible *B. pfeifferi* individuals in the Democratic Republic of Congo (DRC). The mean value of daily cercarial production in the *B. camerunensis*/*S. mansoni* combination (1968.38 cercariae) was higher than in the allopatric combination *B. pfeifferi* from Senegal - *S. mansoni* from Cameroun (386 cercariae) [33], and some sympatric combinations *B. pfeifferi*-*S. mansoni* from Dhofar in (Oman) (464.04 cercariae) [34] and *B. pfeifferi*-*S. mansoni* from Senegal (670 cercariae) [35]. Regarding the transmission of the infection, this ability to shed large numbers of cercariae could balance the disadvantage of a small number of susceptible *S. mansoni* individuals.

The mortality rate is known to be associated with several factors (pathogenicity of the parasite, intrinsic conditions of the snail, experimental conditions) [15]. In our study, the similarity observed in the mortality rates during prepatent and patent periods in both species likely suggests that the parasite is not the only covariate associated with the mortality of snails. The variability of the observed mortality rates between the different populations of both snail species at the end of the experiment would reflect the genetic differences between these populations, and implies a different fitness of the individuals.

### Epidemiological implications

The significantly high infection rates observed in *B. pfeifferi* populations and the long-term survival of *S. mansoni*-infected individuals (> 8 months post-infection) with continued cercarial shedding confirms the predominant role of this species in the transmission of this intestinal parasite in Cameroon. This adaptation of *S. mansoni* to *B. pfeifferi* is likely the result of a good compatibility between the genes of this parasite and its preferential host [22]. Moreover, the low genetic diversity within *B. pfeifferi* populations resulting from the selection of self-fertilization as their main mating system would promote the transmission, maintenance and fixation of susceptibility genes in these populations. The variability of the susceptibility observed in *B. pfeifferi* could explain, at least partly, the differences in prevalence observed in Cameroon. Although the number of infected snails is an important indicator of transmission of infection, the cercarial production by these infested individuals is even more important [36] while evaluating the risk of transmission and extension of schistosomiasis. Thus, infected *B. camerunensis* individuals producing a large number of cercariae might therefore represent a potential risk of increasing *S. mansoni* transmission in Cameroon. In addition, the fact that *B. pfeifferi* snails are becoming scarce in many historical foci could exert a selection pressure enabling a viable adaptation of *S. mansoni* miracidia to *B. camerunensis*. Therefore, disadvantageous environmental changes for *B. pfeifferi* could increase the importance of *B. camerunensis* in the transmission of *S. mansoni*, and thus the incidence of the infection as a consequence of the evolution of the relationship between *Biomphalaria camerunensis* and *S. mansoni*.

## Conclusions

This study confirmed the known predominant role of *B. pfeifferi* in *S. mansoni* transmission in Cameroon and revealed the ability of *B. camerunensis* to efficiently transmit and disseminate this parasite. The ‘vector’ role of this species should therefore be taken into account while assessing schistosomiasis transmission. Special attention and surveillance should be paid to this host since (i) some *B. camerunensis* populations would increase the risk of dissemination of schistosomiasis in Cameroon, and (ii) this intermediate host could represent an important research model to study the immune defense mechanisms against *S. mansoni*. In a context where major hydraulic projects are ongoing in southern Cameroon, together with the important migration flow of humans seeking jobs, malacological studies are highly needed to determine the current distribution of this intermediate hosts and identify susceptible populations to *S. mansoni* throughout the country.

## Additional files

Additional file 1: **Table S1.** Pairwise comparison of the infection rate among snail populations. (DOCX 13 kb)

Additional file 2: **Table S2.** Pairwise comparison of the mortality rate among snail populations. (DOCX 13 kb)

## Abbreviations

G1: First generation
MDA: Mass drug administration
SD: Standard deviation
SIR: Success of infection rate
