# Supplementary material for: Biomphalaria camerunensis as a viable alternative intermediate host for Schistosoma mansoni in southern Cameroon
Source: Parasit Vectors. 2018 Mar 13;11:181. doi: 10.1186/s13071-018-2763-2 (PMC5848459; doi:10.1186/s13071-018-2763-2)
Supplement: Supplementary file 1 — Table S1. Pairwise comparison of the infection rate among snail populations. (DOCX 13 kb) [file 13071_2018_2763_MOESM1_ESM.docx]

**Table S1.** Pairwise comparison of the infection rate among snail populations

| Population | Nkolbisson | Gounougou | Mokolo | Yana Messina | Mounassi | Kede | Sangmelima | Peptonoun |
| --- | --- | --- | --- | --- | --- | --- | --- | --- |
| Nkolbisson | 0  (1) |  |  |  |  |  |  |  |
| Gounougou | 31,91  (<0,0001) | 0  (1) |  |  |  |  |  |  |
| Mokolo | 35,43  (<0,0001) | 0,12  (0,729) | 0  (1) |  |  |  |  |  |
| Yana Messina | 129,57  (<0,0001) | 38,98  (<0,0001) | 35,06  (<0,0001) | 0  (1) |  |  |  |  |
| Mounassi | 128,97  (<0,0001) | 37,65  (<0,0001) | 33,68  (<0,0001) | Fisher Exact  (1,0000) | 0  (1) |  |  |  |
| Kede | 122,72  (<0,0001) | 34,94  (<0,0001) | 31,19  (<0,0001) | Fisher Exact  (0,7440) | Fisher Exact  (1,0000) | 0  (1) |  |  |
| Sangmelima | 152,06  (<0,0001) | 54,84  (<0,0001) | 50,55  (<0,0001) | Fisher exact  (0,0518) | Fisher Exact  (0,0265) | Fisher Exact  (0,0222) | 0  (1) |  |
| Peptonoun | 85,27  (<0,0001) | 11,26  (0,0008) | 8,82  (0,003) | 12,39  (0,0004) | 11,10  (0,0009) | 9,97  (0,0016) | 23,98  (<0,0001) | 0  (1) |

In this table, Chi-Square values are followed by *P*-values are provided in the parenthesis. The blue font indicate significant differences
