# Supplementary material for: Biomphalaria camerunensis as a viable alternative intermediate host for Schistosoma mansoni in southern Cameroon
Source: Parasit Vectors. 2018 Mar 13;11:181. doi: 10.1186/s13071-018-2763-2 (PMC5848459; doi:10.1186/s13071-018-2763-2)
Supplement: Supplementary file 2 — Table S2. Pairwise comparison of the mortality rate among snail populations. (DOCX 13 kb) [file 13071_2018_2763_MOESM2_ESM.docx]

**Table S1.** Pairwise comparison of the mortality rate among snail populations

| Population | Nkolbisson | Gounougou | Mokolo | Yana Messina | Mounassi | Kede | Sangmelima | Peptonoun |
| --- | --- | --- | --- | --- | --- | --- | --- | --- |
| Nkolbisson | 0  (1) |  |  |  |  |  |  |  |
| Gounougou | 0,00  (1,0000) | 0  (1) |  |  |  |  |  |  |
| Mokolo | 5,82  (0,0158) | 4,52  (0,0335) | 0  (1) |  |  |  |  |  |
| Yana Messina | 0,30  (0,5839) | 0,27  (0,6171) | 2,69  (0,101) | 0  (1) |  |  |  |  |
| Mounassi | 3,74  (0,0531) | 3,03  (0,0817) | 14,52  (0,0001) | 4,98  (0,0256) | 0  (1) |  |  |  |
| Kede | 5,45  (0,0196) | 4,52  (0,0335) | 16,99  (<0,0001) | 6,76  (0,0093) | 0,19  (0,6629) | 0  (1) |  |  |
| Sangmelima | 3,58  (0,0585) | 2,87  (0,0902) | 14,42  (0,0001) | 4,81  (0,0283) | 0,00  (1,0000) | 0,26  (0,6101) | 0  (1) |  |
| Peptonoun | 71,47  (<0,0001) | 55,79  (<0,0001) | 29,45  (<0,0001) | 49,41  (<0,0001) | 80,48  (<0,0001) | 83,42  (<0,0001) | 81,34  (<0,0001) | 0  (1) |

In this table, Chi-Square values are followed by *P*-values are provided in the parenthesis. The blue font indicate significant differences
